# A Novel Statistical Optimization Algorithm for Estimating Perfusion Curves in Susceptibility Contrast-Enhanced MRI

**DOI:** 10.3389/fnins.2021.713893

**Published:** 2021-08-26

**Authors:** Zhenghui Hu, Fei Li, Junhui Shui, Yituo Tang, Qiang Lin

**Affiliations:** ^1^Key Laboratory of Quantum Precision Measurement, College of Science, Zhejiang University of Technology, Hangzhou, China; ^2^College of Information Engineering, Zhejiang University of Technology, Hangzhou, China

**Keywords:** intravascular indicator dynamics, gamma-variate function, logarithm linear least square, maximum likelihood estimation, contrast-enhanced MRI

## Abstract

Dynamic susceptibility contrast-enhanced magnetic resonance imaging is an important tool for evaluating intravascular indicator dynamics, which in turn is valuable for understanding brain physiology and pathophysiology. This procedure usually involves fitting a gamma-variate function to observed concentration-time curves in order to eliminate undesired effects of recirculation and the leakage of contrast agents. Several conventional curve-fitting approaches are routinely applied. The nonlinear optimization methods typically are computationally expensive and require reliable initial values to guarantee success, whereas a logarithmic linear least-squares (LL-LS) method is more stable and efficient, and does not suffer from the initial-value problem, but it can show degraded performance, especially when a few data or outliers are present. In this paper, we demonstrate, that the original perfusion curve-fitting problem can be transformed into a gamma-distribution-fitting problem by treating the concentration-time curves as a random sample from a gamma distribution with time as the random variable. A robust maximum-likelihood estimation (MLE) algorithm can then be readily adopted to solve this problem. The performance of the proposed method is compared with the nonlinear Levenberg-Marquardt (L-M) method and the LL-LS method using both synthetic and real data. The results show that the performance of the proposed approach is far superior to those of the other two methods, while keeping the advantages of the LL-LS method, such as easy implementation, low computational load, and dispensing with the need to guess the initial values. We argue that the proposed method represents an attractive alternative option for assessing intravascular indicator dynamics in clinical applications. Moreover, we also provide valuable suggestions on how to select valid data points and set the initial values in the two traditional approaches (LL-LS and nonlinear L-M methods) to achieve more reliable estimations.

## 1. Introduction

Dynamic susceptibility contrast-enhanced magnetic resonance imaging (DSC-MRI) is a useful tool for the quantitative assessment of perfusion-related cerebrovascular parameters in clinical applications (Kosior and Frayne, 2010; Emblem et al., 2014; Arzanforoosh et al., 2021; Jin and Cho, 2021). It requires the injection of a paramagnetic contrast agent. A bolus of the intravascular contrast agent passing through the tissue of interest produces local magnetic-field inhomogeneities that lead to a reduction in the transverse relaxation time (T2*) of the bulk tissue (Rosen et al., 1991; Li, 2014). This susceptibility effect is then recorded in a series of rapidly obtained T2*-weighted gradient-echo images. Converting the signal-time curves into concentration-time (i.e., Δ*R*2^*^(*t*)) curves will yield vascular information associated with tissue perfusion, such as the blood volume, blood flow, and mean transit time (MTT).

However, the concentration-time curve is inevitably contaminated by recirculation of the bolus into the region of interest and residual contrast agent in capillaries. A gamma-variate function has been used to model the first pass of the Δ*R*2^*^(*t*) curve to eliminate these undesired effects (Norman et al., 1981; Davenport, 1983). The original motivation for using this function as an empirical model was its heuristic resemblance to the expected relaxation-rate time-course during the first bolus passage (Thompson et al., 1964; Axel, 1980). The underlying physical connection of the dilution process with the gamma-variate expression has also been clarified in recent studies (Davenport, 1983; Mischi et al., 2008). The selection of an appropriate fitting method is therefore essential when analyzing intravascular indicator dynamics (Perkiö et al., 2002; Pianykh, 2012; Romain et al., 2017; Quarles et al., 2019).

The nonlinear Levenberg-Marquardt (L-M) optimization method is typically used to solve the fitting problem of gamma-variate function since it provides superior accuracy (Benner et al., 1997; Li et al., 2003). However, the use of a nonlinear method requires reliable initial values to guarantee convergence: a moderate deviation from the true values may cause the fit to diverge, and in extreme cases, these errors might be even higher when applying numerical integration over the sample points (Benner et al., 1997). Moreover, nonlinear methods usually suffer from a high computational load. These reasons explain why nonlinear fitting methods have not played a prominent role in clinical applications. A logarithmic linear least-squares (LL-LS) approach (Madsen, 1992; Chan and Nelson, 2004), on the other hand, is more stable and efficient as well as not requiring the initial values to be guessed. The LL-LS method uses a logarithm operation to transforms the original gamma-variate function into an equivalent, linear regression form. Standard linear least-squares fitting is then applied. However, the logarithm operation complicates the statistics of the data, and means that the noise no longer conforms to a Gaussian distribution. As a result, the LS estimator will be biased and the quality of the fitting will be subject to limits imposed by the statistical nature of the data, especially when there are few data or outliers are present.

In a recent study, we needed to determine the regional cerebral blood volume (CBV) on a finer voxel scale throughout the brain in order to produce a more accurate hemodynamic assimilation (Hu et al., 2012; Zhang et al., 2016). Only five or six valid data points were collected in the first-pass phase for perfusion-curve fitting due to the sampling interval being longer than usual. Since the existing two methods were found to perform poorly, it was found to be necessary to develop an alternative approach that combines the advantages of the two methods and still exhibits satisfactory performance even when there are few data points. In this paper, we present a novel statistical optimization method that is well suited to such a real-world problem. We demonstrate that the original perfusion curve-fitting problem can be transformed into a gamma-distribution-fitting problem, by treating the concentration-time curves as a random sample from a gamma distribution with time as the random variable. A robust maximum-likelihood estimation (MLE) algorithm can then be readily applied to solve this problem. The proposed method was evaluated in experiments using both synthetic and real data. Our new method yields physiological information on cerebrovascular dynamics that are more stable and accurate than those obtained using a nonlinear method, while keeping the advantages of the LL-LS method, such as low computational load, and without requiring the initial values to be guessed.

## 2. Derivation of the Relative CBV

**Figure 1:**
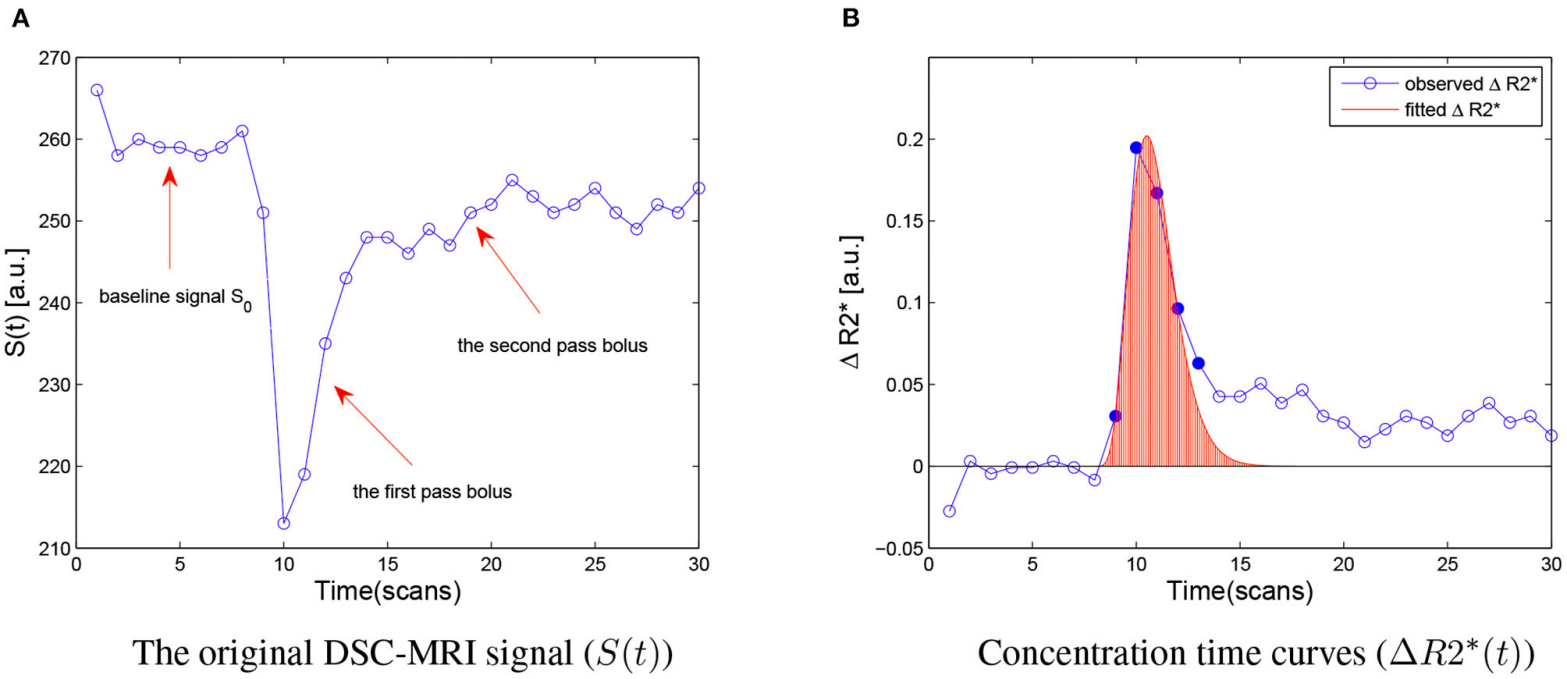
**(A)** The original DSC-MRI signal (*S*(*t*)) obtained from one voxel after administering contrast agent. The baseline signal *S*_0_, the first pass bolus and the second pass bolus are illustrated by the arrow, respectively. **(B)** Concentration-time curves (Δ*R*2^*^) for the same voxel. The Δ*R*2^*^ signal measured during the first bolus pass are shown as filled circles, and other sample points are indicated hollow circles. The red area under the fitted Δ*R*2^*^ curves (red line) is the relative CBV (rCBV) estimated. a.u., arbitrary unit.

DSC MR measures the regional CBV by analyzing changes in the signal intensity after the first pass of a paramagnetic contrast agent. A bolus of intravascular paramagnetic contrast agent passing through the tissue of interest, produces local magnetic-field inhomogeneities that lead to a reduction in the transverse relaxation time T2* of the bulk tissue (Figure 1A) (Rosen et al., 1991). This susceptibility effect is then recorded in a series of rapidly obtained T2*-weighted gradient-echo images. There is an exponential relationship between the relative signal attenuation (*S*(*t*)/*S*_0_) and the local tissue concentration of contrast agent (*C*_*i*_(*t*)) (Rempp et al., 1994):

(1) $$\begin{array}{l} C_{i}\left(t\right)=\frac{-k}{TE}\cdot ln\;\left(\frac{S\left(t\right)}{S_{0}}\right) \end{array}$$

where *k* is an unknown proportionality factor, *TE* is the echo time of the imaging sequences, *S*(*t*) is the MRI signal intensity at time point *t*, and *S*_0_ is the baseline MRI signal intensity before administering the contrast agent.

This relationship can be used to convert the observed signal-intensity-vs.-time curves (*S*(*t*)) into concentration-time curves (Δ*R*2^*^(*t*)) on a voxel-by-voxel basis (Figure 1B). According to the indicator dilution theory, the relative cerebral blood volume (rCBV) is proportional to the area under the Δ*R*2^*^(*t*) curve (Rempp et al., 1994):

(2) $$\begin{array}{l} rCBV=\frac{k}{k_{r}}\cdot \frac{k_{H}}{\rho }\cdot \frac{\int ln\left(S\left(t\right)/S_{0}\right)dt}{\int ln\left(S_{r}\left(t\right)/S_{r_{0}}\right)dt}\cdot \frac{TE}{TE_{r}} \end{array}$$

where *S*_*r*_(*t*)/*S*_*r*_0__ is the relative signal reduction in the reference voxel, ρ = 1.04g/mL is the density of brain tissue, and correction factor *k*_*H*_ accounts for the difference in hematocrit between the voxel of interest and the reference voxel. For our calculations of relative concentration values, these correction factors were set as 1.0.

In practice, the concentration-time curves usually do not only reflect the first bolus pass, but are also affected by a second bolus pass starting 15–20 s after the first one due to the recirculation of the contrast agent bolus. Additionally, residual contrast agent often produces a gradually decreasing bias of the concentration-time curve that is not as steep as the assumed hypothetical exponential decay. For these reasons, the original concentration-time curve can not be used directly for the calculation of vascular parameters. The curve of interest is usually modeled with a gamma-variate function to eliminate these undesired effects (Norman et al., 1981; Davenport, 1983):

(3) $$\begin{array}{l} \Delta R2^{*}\left(t\right)=y=A{\left(t-t_{0}\right)}^{\alpha }e^{-\left(t-t_{0}\right)/\beta } \end{array}$$

where *t*_0_ is the time when the contrast agent is applied in the specified area, and *A*, α, and β are parameters that determine the shape of the function. The rCBV value is proportional to the area (*F*) under the concentration-time curve, and can be calculated as follows (see Appendix A):

(4) $$\begin{array}{l} F=\int_{t_{0}}^{\infty }C_{i}\left(t\right)dt=A\cdot \beta^{\alpha +1}\cdot \Gamma \left(\alpha +1\right) \end{array}$$

## 3. Nonlinear Levenberg-Marquardt Optimization Method

The Levenberg-Marquardt algorithm combines two numerical minimization algorithms: the gradient descent method and the Gauss-Newton method. Specifically, to fit a mode *ŷ*(*t*|*p*) of an independent variable *t* and a vector of *n* parameters ***p*** to a set of *m* data points (*t*_*i*_, *y*_*i*_), the estimation of parameters ***p*** is customary to minimize the sum of the weighted residuals between the known data *y*_*i*_ and the estimated fitting function *ŷ*(*t*|*p*).

(5) $$\begin{array}{l} \chi^{2}\left(p\right)=\overset{m}{\underset{i=1}{\sum }}[\frac{y\left(t_{i}-ŷ\left(t|p\right)\right)}{\sigma_{y_{i}}}{]}^{2}={\left(y-\hat{y}\left(p\right)\right)}^{T}\cdot W\cdot (y-\hat{y}\left(p\right) \end{array}$$

Where σ_*y*_*i*__ denotes the measurement error of *y*(*t*_*i*_). The weighting matrix ***W*** is diagonal with *W*_*i*_*i* = 1/σ_*y*_*i*__, or formally, it can be set to the inverse of measurement error covariance matrix. More generally, ***W*** can also be treated as an optimization parameter. This goodness-of-fit measure is called the chi-squared error criterion (Gavin, 2019). Immediately, the Levenberg-Marquardt algorithm adaptively varies the parameter updates between the gradient descent update and the Gauss-Newton update,

(6) $$\begin{array}{l} \left[W^{T}\cdot T\cdot T+\lambda \cdot I\right]=J^{T}\cdot W\cdot \left(y-\hat{y}\right) \end{array}$$

where *J* is the Jacobian matrix [∂*ŷ*/∂*p*], λ is damping parameter, the parameter update is *h*. Small values lead to a Gauss-Newton update and large values result in a gradient descent update. In Marquardt's update research, the value of λ are normalized to the values of **W**^*T*^ · **T** · **T**, thus,

(7) $$\begin{array}{l} \left[W^{T}\cdot T\cdot T+\lambda \cdot diag\left(W^{T}\cdot T\cdot T\right)\right]=J^{T}\cdot W\cdot \left(y-\hat{y}\right) \end{array}$$

and many variations of the Levenberg-Marquardt method also have been developed (Gavin, 2019). In this paper, the nonlinear Levenberg-Marquardt algorithm was provided by MATLAB's own toolbox.

## 4. Logarithmic Linear Least-Squares Method

Without loss of generality, we assume *t*_0_ = 0. Equation (3) can then be simplified as

(8) $$\begin{array}{l} y\left(t\right)=At^{\alpha }e^{-t/\beta } \end{array}$$

To find time *t*_*max*_ at which the bolus signal peaks, we takes the first derivative of Equation (8) and set it to 0:

(9) $$\begin{array}{l} y^{\prime }\left(t_{max}\right)=At_{max}^{\alpha -1}e^{-t_{max}/\beta }\left(\alpha -\frac{t_{max}}{\beta }\right)\\ \;=0 \end{array}$$

which yields

(10) $$\begin{array}{l} t_{max}=\alpha \beta \Rightarrow \beta =\frac{t_{max}}{\alpha }. \end{array}$$

Substituting β into Equation (8) and letting *t* = *t*_*max*_ leads to,

(11) $$\begin{array}{l} y_{max}=A{\left(\frac{t_{max}}{e}\right)}^{\alpha }\Rightarrow A=y_{max}{\left(\frac{e}{t_{max}}\right)}^{\alpha } \end{array}$$

Equation (8) can now be rewritten in terms of *y*_*max*_, *t*_*max*_, and α:

(12) $$\begin{array}{l} y\left(t/t_{max}\right)=y_{max}e^{\alpha }{\left(\frac{t}{t_{max}}\right)}^{\alpha }e^{-\frac{t}{t_{max}}\alpha } \end{array}$$

Taking the natural logarithm on both sides of Equation (12) produces

(13) $$\begin{array}{l} \log\left(y\left(t^{\prime }\right)\right)=\log\left(y_{max}\right)+\alpha \left(1+\log\left(t^{\prime }\right)-t^{\prime }\right) \end{array}$$

where $t^{\prime }=t/t_{max}$. This equation has the form *y* = *C* + α*x*, when *t*_0_ and *t*_*max*_ are known, *y*_*max*_ and α can be determined from the linear regression of the natural logarithm of the observed values, *y*(*t*′), with (1 + log(*t*′) − *t*′) as the independent variable.

As shown above, the LL-LS method requires additional information about *t*_0_ and *t*_*max*_ to be obtained from the observed data. In practice, *t*_0_ is usually estimated from the time prior to *t*_*max*_ at which *S*(*t*) is within one standard deviation (SD) of the initial baseline signal (Chan and Nelson, 2004), and the location of *t*_*max*_ is found by calculating the centroid of the curve (Madsen, 1992). Although the LL-LS method provides a simplified way of assessing rCBV while avoiding the initial-value problem, and it has already been applied in several clinical applications (Chan and Nelson, 2004; Wu et al., 2004), this method still performs worse than the nonlinear method. This can be due to the logarithm operation complicating the statistics of the recorded data, resulting in the noise no longer conforming to a Gaussian distribution. When sufficient data points are available, the noise can still be treated as an approximate Gaussian distribution (according to the Lévy-Lindeberg Central Limit Theory; see Appendix B) (Casella and Berger, 2001). However, when only a few data points are available or the data are contaminated by outliers, the LS estimates become biased and are no longer characterized by minimum variance. Moreover, the estimation of *t*_*max*_ also introduces extra deviation.

## 5. Proposed Statistical Optimization Method

Assuming *t*_0_ = 0, the first pass of the concentration–time course Δ*R*2^*^(*t*) is rewritten as follows:

(14) $$\begin{array}{l} y\left(t\right)=A_{rCBV}\frac{1}{\beta^{\alpha }\Gamma \left(\alpha \right)}t^{\alpha -1}e^{-t/\beta } \end{array}$$

where *A*_*rCBV*_ is the value of rCBV, $\Gamma \left(\alpha \right)=\int_{0}^{\infty }x^{\alpha -1}e^{-x}dx$ is the gamma function, *t* > 0, α > 0, and β > 0. Note that

(15) $$\begin{array}{l} f\left(t|\alpha ,\beta \right)=\frac{1}{\beta^{\alpha }\Gamma \left(\alpha \right)}t^{\alpha -1}e^{-t/\beta } \end{array}$$

is the probability density function (p.d.f.) of the gamma distribution, and

(16) $$\begin{array}{l} \int_{0}^{\infty }f\left(t|\alpha ,\beta \right)dt=1. \end{array}$$

**Figure 2:**
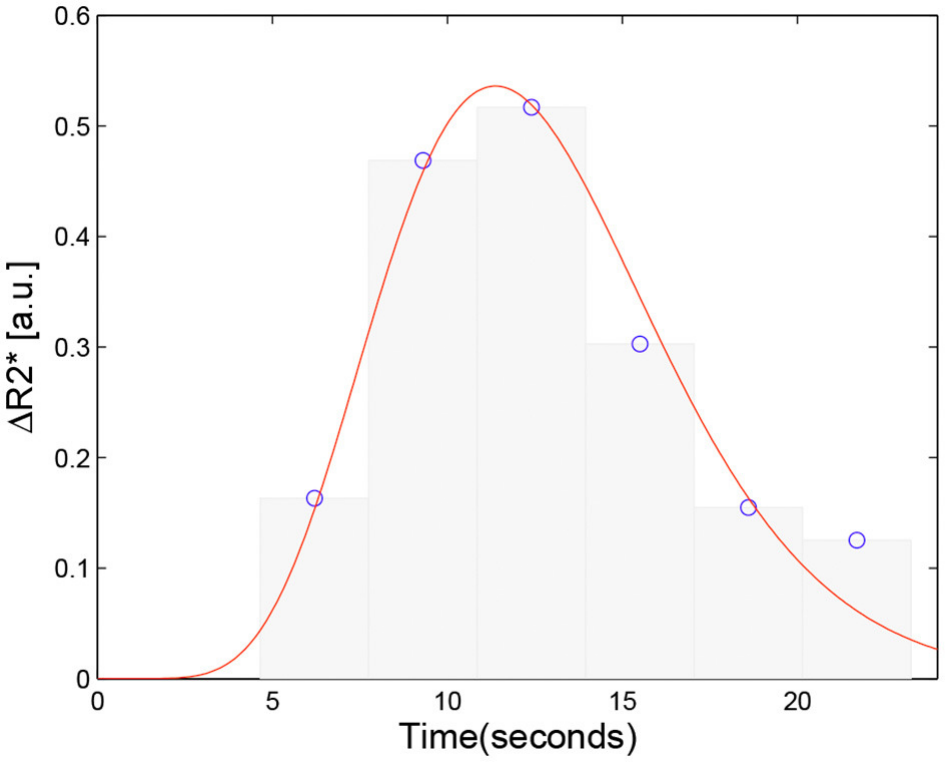
Schematic of the proposed approach. The concentration curve Δ*R*2^*^(*t*) is considered the frequency histogram of a random discrete sample *X* = (*x*_1_, *x*_2_, …, *x*_*n*_) with a gamma distribution *f*(*t* | α, β) associated with the Δ*R*2^*^(*t*) data. The original perfusion curve fitting problem then can be transformed to a gamma distribution fitting problem.

Let *Y* denotes the observed Δ*R*2^*^(*t*) data of the first bolus pass *Y* = (*y*(*t*_1_), *y*(*t*_2_), …, *y*(*t*_*k*_), …), *k* = 1, …, *N* that specifics a gamma distribution *f*(*t* | α, β) with unknown parameters α, and β. Suppose there is a discrete random sample *X* = (*x*_1_, *x*_2_, …, *x*_*n*_) of *n* independent and identically distributed (i.i.d.) observations, which obey the given gamma distribution *f*(*t* | α, β). The random variable *x* may only take *N* different values *t*_1_, *t*_2_, …, *t*_*N*_, and *y*(*t*_*k*_) gives the number of variables obtained in time *t*_*k*_. In other words, the concentration-time curve is considered to be the frequency histogram of the random sample *X* with a gamma distribution *f*(*t* | α, β), where the occurrence frequency of *t*_*k*_ in observation set *X* is proportional to *y*(*t*_*k*_) (Figure 2). This transforms, the original perfusion curve-fitting problem into a gamma-distribution-fitting problem. MLE is a popular choice for estimating parameters of a gamma distribution due to its optimal asymptotic properties (Gupta and Groll, 1961; Harter and Moor, 1965), we therefore applied an MLE approach to solve this problem (Myung, 2003).

Denote *Y* = (*y*(*t*_1_), *y*(*t*_2_), …, *y*(*t*_*k*_), …). The likelihood function is defined as:

(17) $$\begin{array}{l} L\left(\alpha ,\beta \right)|Y)=\overset{n}{\underset{k=1}{\prod }}f\left(y\left(t_{i}\right)|\alpha ,\beta \right) \end{array}$$

Maximizing *L*(α, β|*Y*) with respect to (α, β) is equivalent to minimizing the negative logarithm of *L*(α, β|*Y*), and hence,

(18) $$\begin{array}{l} \log L\left(\alpha ,\beta \right)=-n\alpha \text{ log}\beta -n\log\Gamma \left(\alpha \right)\\ \;+\left(\alpha -1\right)\sum \log x-\frac{1}{\beta }\overset{n}{\underset{k=1}{\sum }}x_{k} \end{array}$$

where the summation is over the *n* sample values.

Assuming that the log-likelihood function is differentiable, it must satisfy the following partial differential equation

(19) $$\begin{array}{l} \{\begin{array}{c} \frac{\partial \left(\log L\left(\alpha ,\beta \right)\right)}{\partial \alpha }=0\\ \frac{\partial \left(\log L\left(\alpha ,\beta \right)\right)}{\partial \beta }=0 \end{array} \end{array}$$

Differentiating Equation (18) we obtain the equations for the MLE:

(20) $$\begin{array}{l} \log\hat{\beta }+\frac{\partial }{\partial \hat{\alpha }}\log\Gamma \left(\hat{\alpha }\right)-\frac{1}{n}\overset{n}{\underset{k=1}{\sum }}\log x_{k}=0. \end{array}$$

(21) $$\begin{array}{l} \hat{\alpha }\hat{\beta }=\frac{1}{n}\overset{n}{\underset{k=1}{\sum }}x_{k} \end{array}$$

Substituting $\hat{\beta }$ in Equation (20) gives

(22) $$\begin{array}{l} \log\alpha -\psi \left(\alpha \right)=\log\bar{x}-\frac{1}{n}\overset{n}{\underset{k=1}{\sum }}\log x_{k} \end{array}$$

where the digamma function $\psi \left(\hat{\alpha }\right)=\frac{\partial }{\partial \hat{\alpha }}\log\Gamma \left(\hat{\alpha }\right)$ is the logarithmic derivative of Γ(α), and $\bar{x}=1/n\overset{n}{\underset{k=1}{\sum }}x\left(t_{k}\right)$ denotes the average of the given data. Equation (22) is implicit in $\hat{\alpha }$, and it is neither possible to find an analytical expression for $\left(\hat{\alpha },\hat{\beta }\right)$ nor to use the Davis tables of ψ-functions directly (Davis, 1933). Masuyama and Kuroiwa prepared tables of $\log\hat{\alpha }-\psi \left(\hat{\alpha }\right)$ for the likelihood solutions of the gamma distribution (Masuyama and Kuroiwa, 1951; Greenwood, 1960).

Furthermore, the second derivatives of the log-likelihoods

(23) $$\begin{array}{l} \{\begin{array}{c} \frac{\partial^{2}\log L\left(\alpha ,\beta \right)}{\partial \alpha^{2}}=\psi_{1}\left(\hat{\alpha }\right)<0\\ \frac{\partial^{2}\log L\left(\alpha ,\beta \right)}{\partial \beta^{2}}=-\frac{\bar{x}}{{\hat{\beta }}^{2}}<0 \end{array} \end{array}$$

where $\psi_{1}\left(\hat{\alpha }\right)=\frac{\partial }{\partial \hat{\alpha }}\psi \left(\hat{\alpha }\right)$, are 1-digamma functions. The second derivatives are negative, and ensure that the log-likelihood function $\log L\left(\hat{\alpha },\hat{\beta }\right)$ is a maximum.

In order to obtain a numerical expression, Thom developed an approximation to the solution (Thom, 1958). The digamma function, $\psi \left(\hat{\alpha }\right)$ has an asymptotic expansion

(24) $$\begin{array}{l} \psi \left(\alpha \right)=\log\alpha -1/\left(2\alpha \right)-\overset{m}{\underset{k=1}{\sum }}{\left(-1\right)}^{k-1}B_{k}/\left(2k\alpha^{2k}\right)+R_{m}, \end{array}$$

where *B*_*k*_ is the Bernoulli number for *B*_1_ = 1/6, *B*_2_ = 1/30, …, and *R*_*m*_ is the remainder after *m* terms.

When α ≥ 1,

(25) $$\begin{array}{l} |R_{m}|\leq \frac{B_{m+1}}{\left(2m+2\right)\alpha^{2m+2}} \end{array}$$

and can be neglected. The approximation is more accurate when α is larger.

For *m* = 1 we have

(26) $$\begin{array}{l} \psi \left(\alpha \right)=\log\alpha -1/\left(2\alpha \right)-1/\left(12\alpha^{2}\right). \end{array}$$

Substituting in Equation (22) yields

(27) $$\begin{array}{l} 12\left(\log\bar{x}-\frac{1}{n}\overset{n}{\underset{k=1}{\sum }}\log x_{k}\right){\hat{\alpha }}^{2}-6\hat{\alpha }-1=0 \end{array}$$

Simplifying by letting $A=\log\bar{x}-\frac{1}{n}\overset{n}{\underset{k=1}{\sum }}\log x_{k}$ produces

(28) $$\begin{array}{l} 12A{\hat{\alpha }}^{2}-6\hat{\alpha }-1=0, \end{array}$$

which is a quadratic equation whose only pertinent root is

(29) $$\begin{array}{l} \hat{\alpha }=\frac{1+\sqrt{1+4A/3}}{4A}. \end{array}$$

Combining this with Equation (21) gives the MLE for the gamma distribution. Thom provided an error table for correcting the estimate obtained from Equation (29) in the case of a small α (Thom, 1958), seen in Table 1.

**Table 1:** Errors for correcting the α estimate.

**$\hat{\alpha }$**	**$\Delta \hat{\alpha }$**	**$\hat{\alpha }$**	**$\Delta \hat{\alpha }$**	**$\hat{\alpha }$**	**$\Delta \hat{\alpha }$**
0.2	0.034	1.0	0.009	1.8	0.004
0.3	0.029	1.1	0.008	1.9	0.003
0.4	0.025	1.2	0.007	2.2	0.003
0.5	0.021	1.3	0.006	2.3	0.002
0.6	0.017	1.4	0.006	3.1	0.002
0.7	0.014	1.5	0.005	3.2	0.001
0.8	0.012	1.6	0.005	5.5	0.001
0.9	0.011	1.7	0.004	5.6	0

*The value of* Δ*α is subtracted from the value of* $\hat{\alpha }$ *obtained from Equation (29)*.

Note that *y*(*t*_*k*_) is a scaled version of the occurrence frequency of *t*_*k*_ in observation set *X*. We have

(30) $$\begin{array}{l} \bar{x}=\frac{\sum_{k=1}^{N}y_{k}t_{k}}{\sum_{k=1}^{N}y_{k}}. \end{array}$$

To summarize, *A*, α, and β are expressed in terms of the observation data *y*_*k*_ and associated time *t*_*k*_ as follows:

(31) $$\begin{array}{l} \{\begin{array}{l} A=\log\frac{\sum_{k=1}^{N}y_{k}t_{k}}{\sum_{k=1}^{N}y_{k}}-\frac{\sum_{k=1}^{N}y_{k}\log t_{k}}{\sum_{k=1}^{N}y_{k}}\\ \hat{\alpha }\hat{\beta }=\frac{\sum_{k=1}^{N}y_{k}t_{k}}{\sum_{k=1}^{N}y_{k}} \end{array} \end{array}$$

## 6. Experimental Validation

In this section, we examine the effectiveness of the proposed approach and compare the performances of the three fitting algorithms based on the concentration-time curve. Because the real values corresponding to the DSC MRI data are unknown, numerical simulations were used to investigate the uncertainties of the estimated cerebrovascular parameters (i.e., rCBV or *F*) for the three fitting algorithms with different signal-to-noise ratios (SNR) and time resolution (Δ*t*) values.

Two subjects participated in this study. Images were acquired with a 1.5-tesla scanner (Signa Excite, GE). The CBV imaging sequence consisted of 30 T2*-weighted images that were collected using an echo-planar gradient-echo (GE) sequence with the following parameters: repetition time = 3, 100 *ms*, echo time = 80 *ms*, flip angle = 90°, field of view = 240 × 240*mm*, matrix size = 128 × 128, and 0.1 mmol/kg Gd-DTPA administered with a power injector. It should be noted that, in order to achieve a sufficient SNR and coverage of the whole brain, the repetition time was increased to be 3.1 s, since the typical value is about 1 s (Zhang et al., 2016).

**Figure 3:**
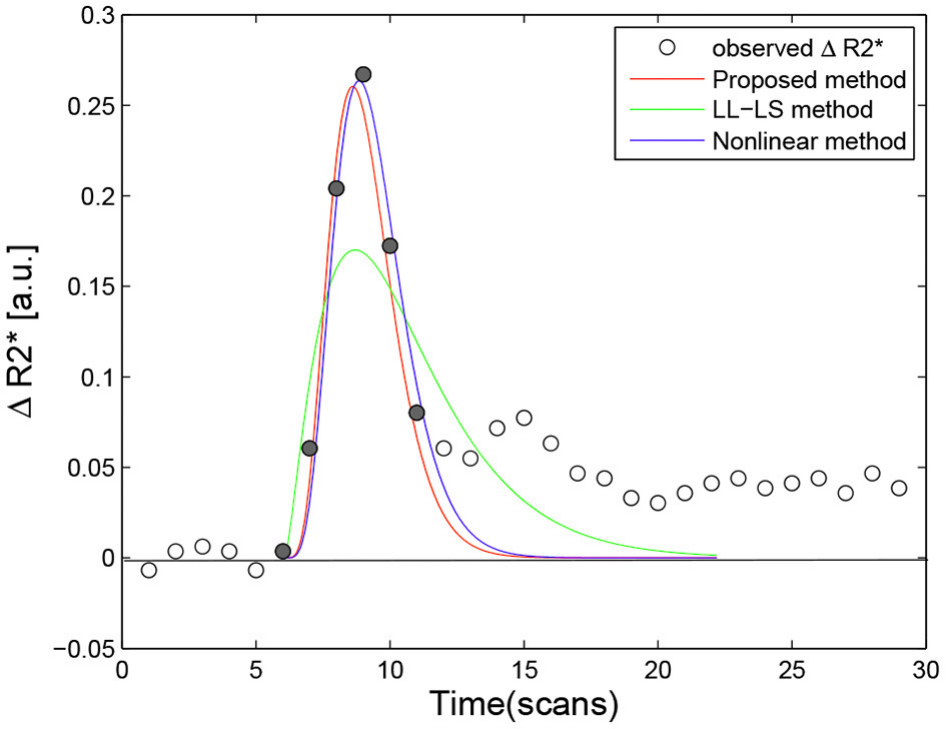
Example of fitting curve obtained using the proposed method (red line, α_*mle*_ = 5.8943, β_*mle*_ = 1.6095, *F*_*mle*_ = 2.4427), the LL-LS method (green line, α_*ls*_ = 2.4364, β_*ls*_ = 5.6789, *F*_*ls*_ = 3.0727), and the nonlinear method (blue line, α_*non*_ = 6.0289, β_*non*_ = 1.7118, *F*_*non*_ = 2.5780) for a single voxel. Filled circles denote valid data points for perfusion-curve fitting; other points are indicate by hollow circles.

Since our method is used to estimate the whole-brain rCBV, involving tens of thousands of data points, we presented only a few examples for a more detailed comparison. Thirty voxels were chosen as the region of interest (ROI) from the two subjects. They were picked from various areas, such as the motor area, visual area, and the thalamus. The concentration-time curves were created for each voxel using (Equation 1). Fitting procedures were applied to these curves using three fitting algorithms (nonlinear L-M method, LL-LS method, and the proposed method), which produced a total of 90 parameter sets {α, β}. Figure 3 gives examples of perfusion-curve fitting for one voxel when using the three methods. The figure demonstrates that all of the parameter sets had a reasonable physiological meaning, and so the three methods could be compared fairly. The ideal concentration-time curves as gamma-variate functions were then generated from these sets with the model

(32) $$\begin{array}{l} \Delta R2^{*}\left(t\right)=\Delta R2^{*}\left(t\right)+K\int_{0}^{t}\Delta R2^{*}\left(t^{\prime }\right)dt \end{array}$$

where *K* = 0.02 is the recirculation weighting factor that describes the recirculation and leakage during the recirculation phase of the curve (Weisskoff et al., 1994). Gaussian-distributed noise was added to Δ*R*2^*^(*t*) at different levels (SNR = 5, 10, 20, 50, and 100) with SNR defined as Chan and Nelson (2004)

(33) $$\begin{array}{l} \text{SNR}=\frac{y_{max}}{\sigma } \end{array}$$

where *y*_*max*_ is the peak of the curves (see Equation 11), and σ is the standard deviation of the added noise. The time resolution Δ*t* was chosen to vary between 0.2 and 3.2 s in steps of 0.2 s. Then the gamma-variate fitting was repeated using the three methods. The onset of the bolus at *t*_0_ was determined by searching from the time point of the maximum back to zero looking for two successive values that were less than a threshold of 10% of the maximum (Benner et al., 1997). The found time points were additionally corrected according to the time resolution and were identical for the three fitting algorithms. Moreover, the estimated parameter {α, β} for the LL-LS method, except where indicated otherwise, was also used as the initial value in the nonlinear fitting procedure.

Two uncertainty values were calculated for the rCBV (i.e., the area under the concentration-time curves) to evaluate the three methods, Benner et al. (1997)

(34) $$\begin{array}{l} \mu_{i}=\frac{\bar{F}-F_{i}}{F_{i}}\cdot 100\% \end{array}$$

and

(35) $$\begin{array}{l} \sigma_{i}=\frac{\sigma_{F}}{F_{i}}\cdot 100\% \end{array}$$

where $\bar{F}$ and σ_*F*_ are the mean and standard-deviation values of rCBV calculated from 250 synthetic curves with parameter set {α_*i*_, β_*i*_}, and *F*_*i*_ is the real rCBV value calculated from Equation (4). Taken overall 90 parameter sets {α, β}, μ gives the percentage of deviation from the correct value and is a measure of how accurately the parameters calculated from the fit agree with the correct ones, and σ is the coefficient of variation as a percentage of all values calculated for *F* and represents a measure of the probability that a calculated value lies within a certain range of the correct value (Benner et al., 1997).

**Figure 4:**
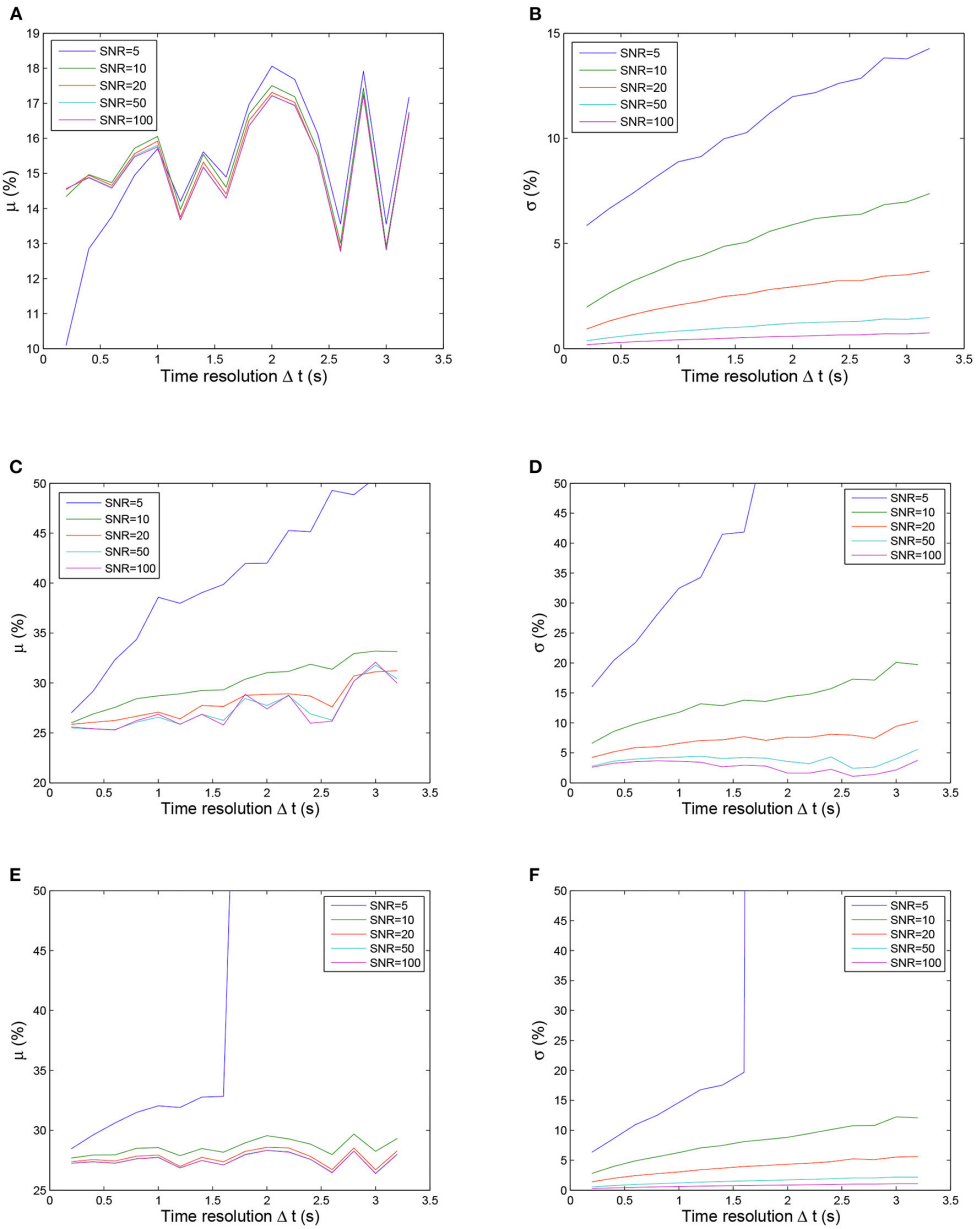
Uncertainty μ (left column) and σ (right column) values calculated using three fitting methods as functions of the time resolution for five SNR values. Note that the ordinate scale is smaller for the proposed method than for other two methods for clarity. The fitting procedure was considered to have failed if the values of the μ and σ uncertainties were larger than 50% of the correct values. From top to bottom: proposed method, LL-LS method, and nonlinear method. **(A)** Uncertainty μ values calculated using the proposed method. **(B)** Uncertainty σ values calculated using the proposed method. **(C)** Uncertainty μ values calculated using LL-LS method. **(D)** Uncertainty σ values calculated using LL-LS method. **(E)** Uncertainty μ values calculated using nonlinear method. **(F)** Uncertainty σ values calculated using nonlinear method.

**Figure 5:**
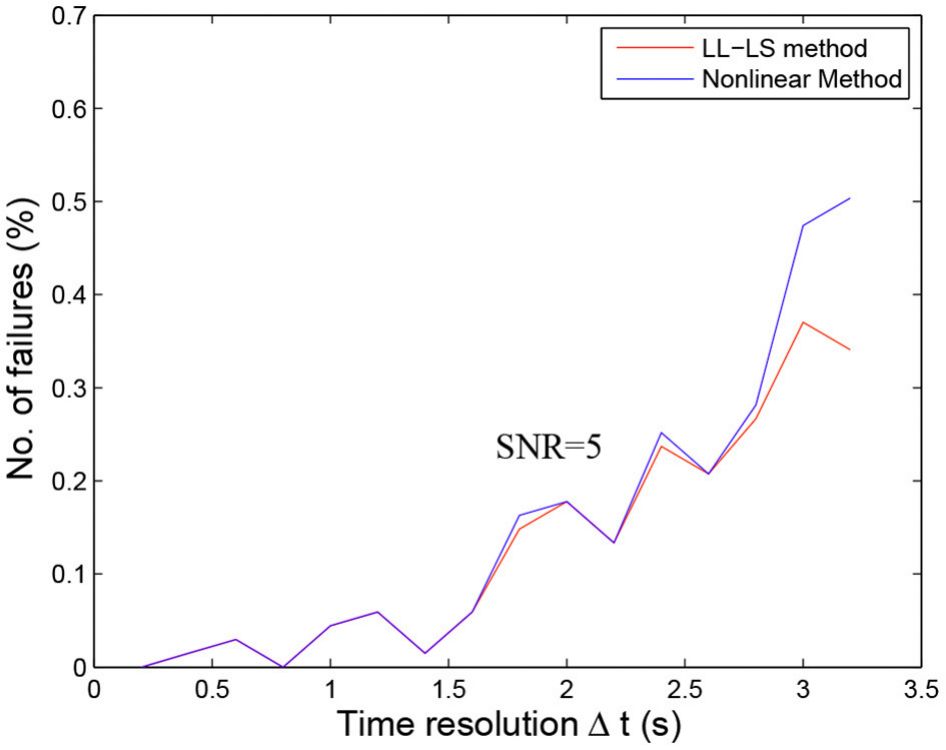
Failures of the fitting procedure with the LL-LS and nonlinear methods as functions of the time resolution (Δ*t*) for SNR = 5. The fitting procedure was considered to have failed if the algorithms did not converge or the calculated values of parameters α and β were imaginary numbers. The estimated parameter {α, β} obtained using the LL-LS method is used as the initial value in the nonlinear fitting procedure. The number of failures of the nonlinear method is thus equal to the number of failures of the LL-LS method plus the number of times that the algorithm for the nonlinear diverged. The number of failures consistently increased as the time resolution decreased. However, there were no failures for the proposed method even for the highest noise level.

Figure 4 shows the calculated uncertainty values for the three fitting methods as functions of the time resolution for the five SNR levels. In general, the quality of the fit depends on the time resolution and the noise level, with the noise having a greater impact on the fitting result when Δ*t* is larger. For all three methods, the uncertainties (μ and σ) of the estimated parameter increased with increasing Δ*t* and with decreasing SNR. The influence of SNR variation was stronger for σ (left panels in Figure 4) than for μ (right panels in Figure 4). The estimated parameter was more stable while Δ*t* decreased and SNR increased (right panels in Figure 4). These observations can be explained by a smaller Δ*t* and larger SNR resulting in more usable sample points with less contamination by noise, thereby achieving more reliable fitting. However, even for the smallest Δ*t* = 0.2 and the lowest noise level (SNR = 100), the estimated parameter deviated from the real values by more than 10% (left panels in Figure 4). This inherent deviation can contribute to the additional effect of contrast agent recirculation in the simulation. In all situations, the nonlinear method (Figures 4E,F) performed better than the LL-LS method (Figures 4C,D), while the proposed approach (Figures 4A,B) exhibited far superior stability and accuracy than the other two methods. While both the conventional LL-LS and nonlinear methods delivered decent estimates when the SNR was large (Figure 4), they led to a failure of the calculated parameters for the lowest SNR (SNR = 5) and the median Δ*t* case (Δ*t* > 1.6 s) where the uncertainties of the estimated parameter were larger than 50% (blue lines in Figures 4C–F). In contrast, the proposed approach generated significantly better results for different time resolutions and noise levels, illustrating the benefits of transforming the original curve-fitting problem into a distribution-fitting problem. Moreover, the fitting procedure for the LL-LS and nonlinear methods failed for the largest time resolution (Δ*t* = 3.2 s) and the highest noise level (SNR = 5). Figure 5 shows the failure rate of the fitting procedure as a function of the time resolution (Δ*t*). Not surprisingly, the number of failures increased with increasing Δ*t*, demonstrating that obtaining more sample points during the first pass of the contrast agent can help to build a more accurate view of the vascular indicator dynamics (Lu and Monahan, 1993).

## 7. Discussion and Conclusion

Indicator dilution analysis involves computing perfusion-related parameters (e.g., blood volume, blood flow, and mean transit time) from the observed flow of a contrast agent passing through the vascular system. This analysis method is applied in a diverse range of medical fields. The measurements might yield valuable diagnostic information for use in various applications, such as the assessment of tissue perfusion, the detection of ischemic regions and cancer hypervascularization (Ruediger et al., 1964; Eckersley et al., 2002; Yang et al., 2003). The available measurement methods extend beyond DSC-MRI, to include other bolus-tracking imaging methods, such as contrast-enhanced computed tomography (Pienn et al., 2013), contrast-enhanced ultrasound imaging (Feinstein, 2004), and scintigraphy (Anger, 1964). In this paper, we have proposed a novel statistical optimization strategy for perfusion analysis, with the initial motivation being that the voxel-wise evaluation of the rCBV information needs to be performed from a series of DSC-MRI observations in a real clinical application (Zhang et al., 2016). By designing the concentration-time curves as a random sample from a gamma distribution with time as the random variable, we have transformed the original perfusion curve-fitting problem into a gamma-distribution-fitting problem; MLE is then adopted to solve this problem. Since real values from a real experiment were not available, we used simulation experiments to compare the proposed method with conventional methods. The obtained simulation results have demonstrated that the proposed strategy has the potential to provide a more stable and accurate perfusion analysis than a conventional curve-fitting strategy for different time resolutions and noise levels.

We consider that the improved performance of the proposed method is attributable to the reasons given below. The LL-LS method implicitly assumes that log*y*(*t*) conforms to a Gaussian distribution with the right-hand side (RHS) of Equation (13) as its mean and a constant variance. Hence, the small fitting error near *t* = *t*_0_ (we assume *t*_0_ = 0), that is, at *y*(*t*_0_) = 0, could cause a large error at log*y*(*t*_0_). Then, the large change in the left-hand side (LHS) of Equation (13) results a larger error in the estimation of α than for the LS solution based on Equation (13). Unlike the LL-LS method, the proposed method considers that all the observations *y*(*t*_*k*_), *k* = 1, …*N* are independent samples from a gamma distribution (Equation 15) with unknown parameters α and β. The MLE method estimates those two parameters by maximizing the probability of obtaining the observed data. Moreover, compared with the other two methods, the LL-LS method requires additional information on *t*_*max*_ that also increases the deviation.

**Figure 6:**
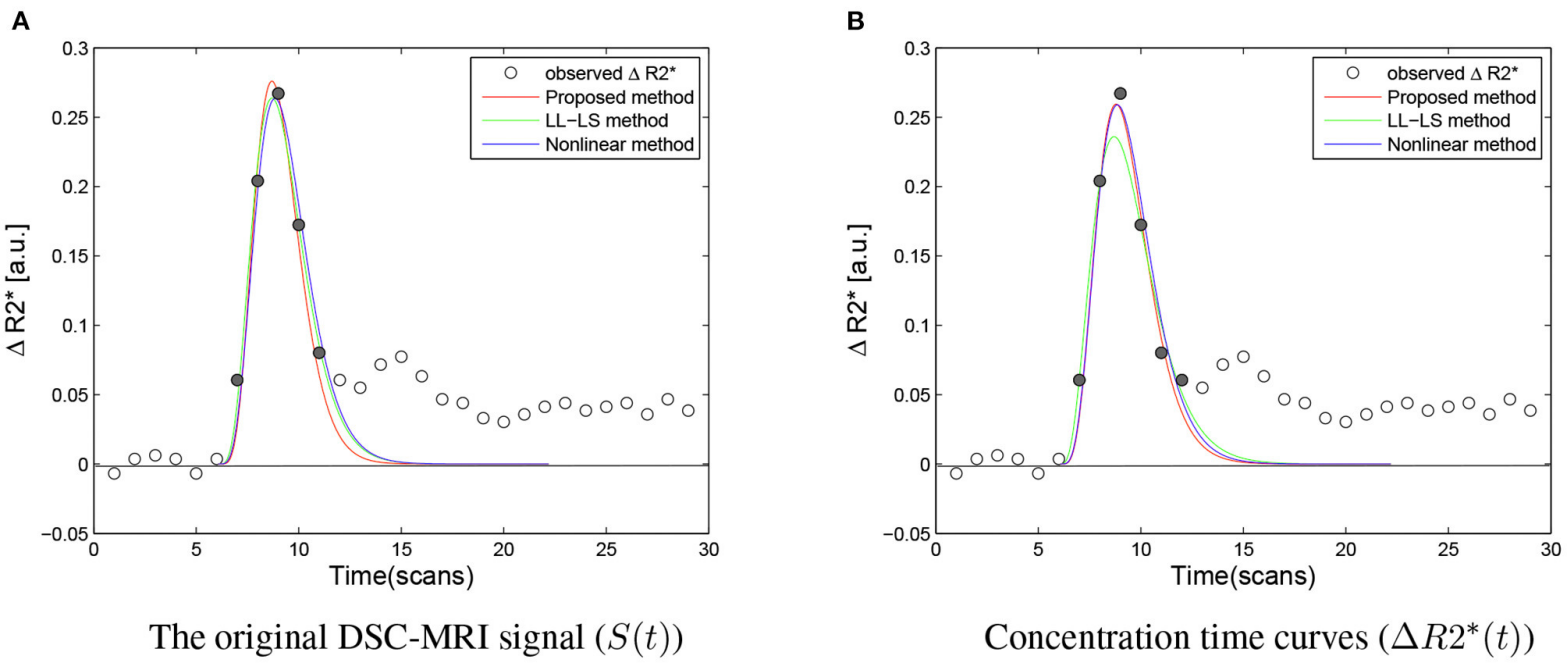
**(A)** Compared with Figure 3, when the first point near to *t* = *t*_0_ was excluded from the fitting, the three methods produced very similar results, illustrating that this point have greater computational weight than other point in the LL-LS method (α_*mle*_ = 6.9087, β_*mle*_ = 1.3795, *F*_*mle*_ = 2.4313, α_*ls*_ = 5.5731, β_*ls*_ = 1.7838, *F*_*ls*_ = 2.5687, α_*non*_ = 6.0294, β_*non*_ = 1.7116, *F*_*non*_ = 2.5780). **(B)** In comparison with the first point near to *t* = *t*_0_, including the additional last point hardly influenced the results. The nonlinear method and the proposed method showed relatively stable performances for the choice of data points due to all observations being treated in an equal manner (α_*mle*_ = 5.8765, β_*mle*_ = 1.7348, *F*_*mle*_ = 2.6190, α_*ls*_ = 4.4527, β_*ls*_ = 2.3626, *F*_*ls*_ = 2.6610, α_*non*_ = 5.6077, β_*non*_ = 1.8767, *F*_*non*_ = 2.6624). The Δ*R*2^*^ signal obtained during the first bolus pass is shown as filled circles, while the other sample points are indicated by hollow circles. The area under the fitted Δ*R*2^*^ curve corresponds to the estimated rCBV.

Figure 6 provides a comparison of the three methods for different data sets. When the data point near *t* = *t*_0_ was excluded from the fitting, the LL-LS method obtained estimates similar to those from the other two methods, illustrating the great computational weight for this data point (compare green lines in Figures 3, 6A). In contrast, including the data point far from *t* = *t*_0_ had little influence on the results (compare green lines in Figures 3, 6B). Because both the nonlinear and proposed methods minimize the estimation error averaged over all observations, they still show relatively stable estimates irrespective of the choice of data points (Figure 6B). This is particularly important for the voxel-wise analysis in the whole brain where it is impossible to check all the data points. The width of the first bolus pass shows a wide variation due to different imaging regions and different molarity/dosages for the contrast agents (Wirestam et al., 2009; Schmiedeskamp et al., 2013). This situation may result in valuable data points being missed or extra data points being included. Our algorithm exhibits more stable and accurate performance in all simulation situations, and hence it has the potential to cope better with undesirable aspects of the image acquisition, such as the inclusion of improper data points, unforeseen data errors, poor image alignment, and the use of new protocols/tracers.

Our study also provides suggestions on how to achieve more reliable estimates when using the two conventional methods in practical applications. As discussed above, the data point near *t* = *t*_0_ (we suggest *t* < 1 s) should be excluded from the fitting when using the LL-LS method. Moreover, compared with the previous empirical approach for the initial value in the nonlinear method, using estimates from the LL-LS method as the initial values in the nonlinear method can reduce the probability of failure for the fitting procedure by about 10-fold (Benner et al., 1997).

Different from a previous method (Scalzo and Liebeskind, 2016), the parameters were computed using a bolus tracking method based on the deconvolution of the time-density curve on a pixel-by-pixel basis. At first, our method was proposed to be applied to the specific situation of estimating the whole brain rCBV curve (Zhang et al., 2016), which means fitting the perfusion curve by involving only a few data points. As we mentioned above, in the case of a small sample, our proposed strategy can also ensure a more accurate and stable performance. On the other hand, for the parameter estimation, although his paper uses the EM algorithm, which is based on the MLE principle. But in their M-step, they adopt a numerical optimization technique. This iterative strategy can approach the optimal solution well, but it may increase the time cost. Then they used the Bayesian Information Criterion (BIC) to determine the optimal K. As we have known that BIC requires a certain amount of data to ensure accuracy, and insufficient data points will lead to inaccurate estimation. Compared with this numerical optimization, we adopt an approximate asymptotic expansion to determine the optimal parameter. Specifically, according to an approximation that Thom developed to the solution, the digamma function, $\psi \left(\hat{\alpha }\right)$ has an asymptotic expansion (Thom, 1958). Therefore, this approximate solution our proposal adopts will allow us to quickly determine the parameters, greatly saving the time cost, and there is no need to manually set the solution procedures.

Furthermore, the proposed strategy could potentially be improved by constructing several bias-correction forms of the MLE estimator to improve the optimal properties of the original MLE in the case of small samples (Choi and Wette, 1969; Giles and Feng, 2009). Also, the strategy could be extended to an estimation scheme containing location parameter *t*_0_ for the three-parameter gamma distribution (Cohen and Whitten, 1982; Anger, 1995). In addition, it is possible to accommodate other mathematical models and include prior knowledge from other modalities so as to further improve the performance (Wu et al., 2004). While it is always possible to transform a distribution-fitting problem into a curve-fitting problem by fitting a curve to a histogram, it is rarely possible to perform the reverse procedure. However, in this paper, we have presented such an example. Despite its success, several questions related to the mathematics underlying the approach still need to be clarified, which will limit our proposal to some extent in explaining the underlying mechanism, such as the optimal asymptotic properties of the MLE estimator for discrete sampling, and the relationship between time resolution and sample size.

In conclusion, the results reported here have demonstrated that the proposed strategy exhibits attractive performance relative to the conventional methods. The algorithm is easy to implement, and it is equivalent to the LL-LS method in terms of computational cost *O*(*n*), and it dispenses with the need to guess the initial values. Our method involves transforming the curve-fitting problem into the probability distribution fitting problem, and the perfusion curve can be well fitted by the MLE estimator. Thus, it is not only suitable for contrast-enhanced MRI but also other methods of bolus-tracking perfusion imaging, for example, X-rays, CT, PET, etc. We therefore suggest that the proposed strategy could represent an alternative option for assessing intravascular indicator dynamics from bolus-tracking perfusion imaging. Currently, the method is successfully applied to our study on hemodynamic assimilation (Zhang et al., 2016).

## Data Availability Statement

The raw data supporting the conclusions of this article will be made available by the authors, without undue reservation.

## Ethics Statement

The studies involving human participants were reviewed and approved by College of Science, Zhejiang University of Technology. The patients/participants provided their written informed consent to participate in this study.

## Author Contributions

ZH conceived and designed the experiments, performed the experiments and analyzed the data, and wrote the manuscript. ZH, FL, and QL edited and approved the final version of the manuscript. All authors contributed to the article and approved the submitted version.

## Conflict of Interest

The authors declare that the research was conducted in the absence of any commercial or financial relationships that could be construed as a potential conflict of interest.

## Publisher's Note

All claims expressed in this article are solely those of the authors and do not necessarily represent those of their affiliated organizations, or those of the publisher, the editors and the reviewers. Any product that may be evaluated in this article, or claim that may be made by its manufacturer, is not guaranteed or endorsed by the publisher.

## A. Calculating rCBV From Γ-Variate Function

The area *F* under the concentration time curve is proportional to the blood volume, and can be calculated as follows:

(A1) $$\begin{array}{l} F=\int_{t_{0}}^{\infty }C_{i}\left(t\right)dt\\ \;=\int_{t_{0}}^{\infty }A\cdot {\left(t-t_{0}\right)}^{\alpha }e^{-\left(t-t_{0}\right)/\beta }dt\\ \;=\int_{0}^{\infty }A{t^{\prime }}^{\alpha }e^{-t^{\prime }/\beta }dt^{\prime }\;\left(t^{\prime }=t-t_{0}\right)\\ \;=A\cdot \beta^{\alpha +1}\int_{0}^{\infty }(\frac{t^{\prime }}{\beta }{)}^{\alpha }e^{-t^{\prime }/\beta }d\left(\frac{t^{\prime }}{\beta }\right)\\ \;=A\cdot \beta^{\alpha +1}\cdot \Gamma \left(\alpha +1\right) \end{array}$$

## B. Lévy-Lindberg Central Limit Theory

Let *X*_1_, *X*_2_, …be a sequence of iid random variables with *EX*_*i*_ = μ and 0 < Var $X_{i}=\sigma^{2}<\infty$. Define ${\bar{X}}_{n}\;=\;\left(1/n\right)\overset{n}{\underset{i=1}{\sum }}X_{i}$. Let *G*_*n*_(*x*) denote the cumulative distribution function (cdf) of $\sqrt{n}\left({\bar{X}}_{n}-\mu \right)/\sigma$. Then, for any *x*, −∞ < *x* < ∞,

(B1) $$\begin{array}{l} \underset{n\to \infty }{\lim}G_{n}\left(x\right)\;=\;\int_{-\infty }^{x}\frac{1}{\sqrt{2\pi }}e^{-y^{2}/2}dy; \end{array}$$

that is, $\sqrt{n}\left({\bar{X}}_{n}-\mu \right)/\sigma$ has a limiting standard normal distribution (Casella and Berger, 2001).
